# Selection of Conserved Epitopes from Hepatitis C Virus for Pan-Populational Stimulation of T-Cell Responses

**DOI:** 10.1155/2013/601943

**Published:** 2013-11-21

**Authors:** Magdalena Molero-Abraham, Esther M. Lafuente, Darren R. Flower, Pedro A. Reche

**Affiliations:** ^1^Facultad de Medicina, Sección de Inmunología, Universidad Complutense de Madrid, Avenida Complutense S/N, 28040 Madrid, Spain; ^2^School of Life and Health Sciences, University of Aston, Aston Triangle, Birmingham B4 7ET, UK

## Abstract

The hepatitis C virus (HCV) is able to persist as a chronic infection, which can lead to cirrhosis and liver cancer. There is evidence that clearance of HCV is linked to strong responses by CD8 cytotoxic T lymphocytes (CTLs), suggesting that eliciting CTL responses against HCV through an epitope-based vaccine could prove an effective means of immunization. However, HCV genomic plasticity as well as the polymorphisms of HLA I molecules restricting CD8 T-cell responses challenges the selection of epitopes for a widely protective vaccine. Here, we devised an approach to overcome these limitations. From available databases, we first collected a set of 245 HCV-specific CD8 T-cell epitopes, all known to be targeted in the course of a natural infection in humans. After a sequence variability analysis, we next identified 17 highly invariant epitopes. Subsequently, we predicted the epitope HLA I binding profiles that determine their potential presentation and recognition. Finally, using the relevant HLA I-genetic frequencies, we identified various epitope subsets encompassing 6 conserved HCV-specific CTL epitopes each predicted to elicit an effective T-cell response in any individual regardless of their HLA I background. We implemented this epitope selection approach for free public use at the EPISOPT web server.

## 1. Introduction

 Hepatitis C virus (HCV) causes a chronic infection that produces liver fibrosis (20% of infected individuals) which can lead to liver failure and the development of liver carcinomas [1]. An estimated 170 million people (3% of the world population) are HCV carriers. Moreover, the prevalence of the infection and associated complications are increasing [1]. Standard therapy against HCV consists of a combination of interferon and ribavirine. This treatment improves symptoms and survival prognosis of patients infected with HCV. However, 50% of patients do not respond adequately, with genotype 1 being especially refractory to treatment [2]. Thus, the development of alternative treatments against HCV, including an effective vaccine, is of paramount importance.

No vaccine is currently available for HCV infection [3]. With the increasing discovery of new anti-HCV small molecule drugs, some may argue that liver disease associated with HCV will largely disappear and thus that HCV vaccine discovery will become superfluous. However, few of the 170+ million HCV infected population will have access to costly medications. Nor will these medicines treat all patients with equal effectiveness. Therefore, prophylactic treatment of HCV infection by a vaccine represents a cost effective and efficient alternative to medicines. Intravenous drug users—the major group suffering new HCV infections in most locations—have an increased likelihood of reinfection and would thus also benefit from prophylactic treatments able to prevent chronic HCV infection.

 HCV exhibits extraordinary genetic diversity, including at least 6 genotypes (1 (a, b, c), 2 (a, b, c), 3 (a, b), 4a, 5a, and 6a) and 52 different subtypes [2, 4], and this represents a major challenge for vaccine development. Work in humans and chimpanzees (the only animal model susceptible to HCV) shows that antibodies are not decisive in resolution and control of HCV infection [5, 6] possibly because this response is focused on regions of the virus envelope, which experiences extreme sequence variability. In contrast, there is evidence that T cells play a fundamental role in the control of HCV infection, since viral clearance observed during acute transient infection correlates with the activation of CD4 T helper (Th) lymphocytes and, importantly, with a broad and sustained response by cytotoxic CD8 T lymphocytes (CTLs) [7, 8]. In contrast, these responses are weak and limited in patients with chronic infection [5, 9–13]. CTLs contribute to the containment of viral infection by eliminating viral-infected cells and indirectly by secretion of antiviral factors [14], while CD4 Th cells cooperate to produce cytokines that stimulate effector functions of CTLs [15]. Together, this suggests that the development of a vaccine against HCV will likely rely on the induction of T-cell responses and in particular a CTL response. It has been shown that it is possible to induce potent and specific CTL responses when immunizing with adenoviral vectors encoding HCV products [16] and to protect chimpanzees from HCV infection [17]. However, given the extreme mutability of HCV, it is difficult to conceive that acquired immunity to a single HCV strain could protect against other, clearly divergent, strains [18] or prevent the emergence of mutants that escape immune responses [19, 20].

CTL responses are directed against short peptides, epitopes, presented by human leukocyte antigens (HLA I) expressed on the cell surface of antigen presenting and target cells. Therefore, HCV immune evasion could be avoided by appropriate selection of conserved HCV-specific CTL epitopes. Currently, there are hundreds of *bona fide* HCV-specific CTL epitopes deposited in specialized databases. However, HCV diversity and extreme polymorphism of HLA I molecules restricting the CTL responses make optimal epitope selection anything but straightforward, particularly if our aim is developing widely protective epitope-based vaccine. For such a task, we started with a set of 245 HCV-specific CTL epitopes, known to be targeted in the course of a natural infection, and sought minimal subsets of invariant epitopes that could be basis for a widely protective epitope-based vaccine. We found various epitope subsets encompassing 6 conserved HCV-specific CTL epitopes that we predicted could elicit an effective T-cell response in any individual regardless of their HLA I background. Here, we analyzed these results and discussed the reason for epitope conservation. We also introduced EPISOPT (http://imed.med.ucm.es/Tools/episopt.html), a server that implements our approach for selecting epitope combinations providing the largest population protection coverage.

## 2. Materials and Methods 

### 2.1. HCV-Specific CTL Epitopes

We used the EPIMHC [21] and IEDB [22] databases to identify HCV-specific CTL epitopes. Specifically, we collected a set of 245 peptide sequences comprising HCV-specific CTL epitopes. These peptides were reported to stimulate the response of human CTLs elicited in the course of a natural infection by HCV in humans and all have nine residues. When available, we also collected the human MHCI molecule that was determined to restrict the CTL response against the peptide. This peptide set is provided as Supplementary Material in Additional File S1 available online at http://dx.doi.org/10.1155/2013/601943 and will be also provided by the corresponding author upon writing request.

### 2.2. Retrieval, Processing, and Multiple Sequence Alignment of HCV Polyproteins

 We generated a multiple sequence alignment (MSA) encompassing 684 entire HCV-polyproteins using MUSCLE [23] with default settings. The HCV-polyproteins included representatives from all HCV genotypes (Table 1). We obtained them from the translation feature (CDS) of HCV nucleotide records retrieved after *GenBank* genomic accessions identified in the European HCV database (EuHCVdb). To ensure working with full-length polyproteins, we only considered translated CDS with more than 3000 amino acid residues. The multiple sequence alignment (MSA) is provided as supplementary material in Additional File S1.

### 2.3. Generation of an HCV-Reference Sequence with the Variables Sites Masked

 We generated a HCV-reference sequence by calculating the sequence variability in the MSA of HCV-polyproteins using the Shannon entropy [24], *H*, as a variability metric [25–27]. Briefly, the Shannon entropy is given by

(1) $$\begin{array}{c} H=-\sum_{i=1}^{M}P_{i}\text{lo}{\text{g}}_{2}P_{i}\text{,} \end{array}$$

where *P*_*i*_ is the fraction of residues of amino acid type *i* and *M* is equal to 20, the number of amino acid types. *H* ranges from 0 (total conservation, only one amino acid type is present at that position) to 4.322 (all 20 amino acids are equally represented in that position).

 We assigned the sequence variability, *H*, to the HCV-polyprotein obtained from *GenBank* accession M62321 (protein id: AAA45676.1) and subsequently masked all positions with a variability, *H*, greater than 0.5, ensuring that the remaining residue positions are highly conserved [28, 29]. 

### 2.4. Prediction of Epitope Presentation by HLA I and Computation of Population Protection Coverage (PPC)

 CTL epitopes are peptides presented by HLA I molecules and recognized by cytotoxic CD8 T lymphocytes (CTLs). For HLA I molecules can only present to T cells those peptides that bind to them, we anticipated HLA I presentation of epitomes/peptides by predicting HLA I binding. Specifically, we used 55 HLA I-specific motif position-specific scoring matrices (PSSMs) [30–32] to predict epitope binding to the corresponding HLA I molecules. We considered that a given peptide binds to specific HLA I molecule when its binding score ranks within the top 3% percentile of the binding scores computed for 1000 random 9mer peptides (average amino acid composition of proteins in the SwissProt database).

The population protection coverage (PPC) of an arbitrary set of CTL epitopes is given by the proportion of the population that could potentially mount an immune response to any of these epitopes and can be computed by knowing the gene frequencies of the HLA I alleles that can present the epitopes. Given the required HLA I restriction of CTL responses, the PPC for a set of CTL epitopes matches the proportion of the population that exhibits at least one of the HLA I alleles that can bind and present any of those epitopes, which corresponds to the cumulative phenotype frequency (CPF) of the targeted HLA I alleles. In this work, we computed CPF using the equations described elsewhere in [26] and the HLA I allelic and haplotype frequencies published by Cao et al. [33] for 5 major American ethnic groups (Black, Caucasian, Hispanic, Native American, and Asian). HLA I allele frequencies published by Cao et al. [33] cover most common HLA-A, -B, and -C alleles; ~10–20% of all available HLA I sequences in IMGT/HLA database [34].

### 2.5. Identification of Optimal Epitope Combinations with a Given Population Protection Coverage

 In order to identify minimal sets of epitopes (optimal epitope combinations) with a target PPC within a starting set of CD8 T-cell epitopes, we first find the HLA I molecules that could present those epitopes by predicting their binding to 55 HLA I molecules (HLA I binding profile), as indicated elsewhere in Section 2. With the predicted HLA I binding profiles, we can compute CPF and, hence, epitope PPC. Once we have the epitope HLA I binding profiles, we search for epitope combinations from the initial set of epitopes, starting with one epitope and iteratively increasing the number of epitopes being considering, which reach the target PPC. At each iteration, we also computed the largest PPC that can be reached with the number of epitopes being considered. Because PPC vary for the 5 ethnic groups for which we have HLA I gene allele frequencies, we seek that the target PPC is reached in each ethnic group. We made this method available for identifying minimal set of epitope combinations reaching a target PPC through the EPISOPT web server. EPISOPT is implemented on an Apache Web server running under the Mac OSX operating system. The EPISOPT functional core consists of a PERL CGI (Common Gateway Interface) script that handles the input, executes the above outlined methods, and then assembles and displays the results.

### 2.6. Other Procedures

We identified three-dimensional (3D) structures of HCV proteins upon BLAST searches [35] with the epitope sequences against the PDB sequence database at NCBI (http://blast.ncbi.nlm.nih.gov/Blast.cgi). We obtained solvent accessibility from the relevant 3D structures using the program NACCESS [36], and we used the server MAST (http://imed.med.ucm.es/Tools/msat.html) for mapping the accessibility onto the 3D structures.

## 3. Result and Discussion

 HCV vaccines could prevent chronic HCV infection, a leading cause of both liver fibrosis and liver cancer. Unfortunately, unlike hepatitis A virus (HAV) and hepatitis B virus (HBV) [37, 38], no such HCV vaccine exists. Most vaccines pursue the generation of long lasting immunity mediated by neutralizing antibodies. Thus, current vaccines for HAV and HBV are based on viral-specific recombinant proteins eliciting such type of immunity [38, 39]. In contrast, immunization trials with recombinant HCV proteins have all failed [37], likely because antibodies do not play a key role in the control of the infection by HCV [5, 6]. Targeting the cellular arm of the adaptive immune system residing in the T cells is surely the key for the development of a successful HCV vaccine [7, 8], and multiple T-cell epitope-based vaccines are particularly suitable for that task.

**Figure 1:**
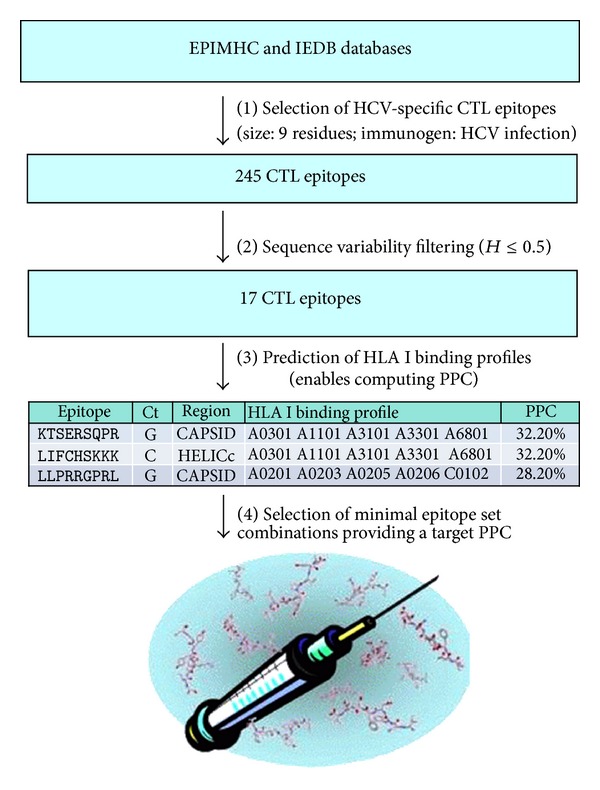
HCV epitope selection approach. Overview of the approach devised to select invariant HCV-specific CTL epitopes that could be the basis for a broadly protective epitope-based vaccine against HCV. The approach consists of 4 basic steps: (1) selection of epitopes from databases according to the indicated criteria. Immunogen HCV infection means that all selected HCV epitopes were reported to be targeted in the course of a natural infection in humans. They are immunogenic. (2) Sequence variability filtering. We only considered epitopes that do not contain any residue with a variability given by Shannon entropy, *H*, greater than 0.5. (3) Prediction of epitope HLA I binding profiles which enables computation of the percentage of the population that will be able to elicit a T-cell response against a particular epitope (PPC). (4) Selection of subsets of epitopes that with a minimum number of epitopes reach a target PPC.

 HCV diversity and HLA polymorphisms are a handicap for developing a broadly protective T-cell epitope-based vaccine against HCV. Therefore, epitope selection is a key step for epitope-vaccine design. Given the relevance of CTL responses in the containment of HCV acute infection [7, 8], in this work we sought to find invariant HCV-specific CTL epitopes that could serve to develop approach depicted in Figure 1.

 A key feature of the approach is the use of already discovered CTL epitopes. Previous work has yielded large numbers of HCV-specific epitopes, which are available in specialized databases such SYFPEITHI [40], JenPep [41], and MHCBN [42], TEPIDAS [43], Immune Epitope database [44], and EPIMHC [21]. In our study, we collected the HCV-specific epitopes from the EPIMHC and Immune Epitope databases. We sought published, annotated epitopes that are targeted in the course of a natural HCV infection and a size of nine residues, assembling a set of 245 HCV-specific CTL epitopes (provided as supplementary material in Additional File S1). The bases for the selection criteria are the following. Appropriate antigen processing is a prerequisite for HLA presentation and limits T-cell recognition [45, 46]. By considering CTL epitopes that are targeted in the course of a natural HCV infection, we assumed that (A) antigen processing is conserved (it will be futile to elicit a T-cell response against an epitope that it is not processed and available for presentation in the course of an infection with HCV) and (B) epitope presentation and T-cell recognition is only determined by HLA I binding. As for the size of the epitopes, HLA I molecules bind and present peptides from 8 to 11 residues but most of them have 9 residues [30]. There are reports in the literature echoed in the databases of much large peptides, up 30 amino acids, that are capable of eliciting CD8 T-cell responses. These peptides do not represent optimal CD8 T-cell epitopes, as they need further processing prior to HLA I binding.

**Figure 2:**
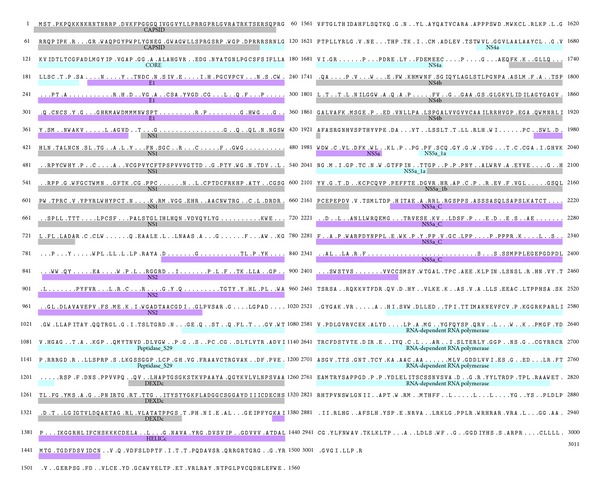
HCV polyprotein with variable positions masked. The figure shows the amino acid sequence HCV-polyprotein (protein id; AAA45676.1) with residues positions with a sequence variability >0.5 masked (shown as dots). We computed sequence variability from a multiple sequence alignment of HCV polyproteins using Shannon entropy, *H*, and masked the variable sites on the reference sequence as detailed in Section 2. The mature proteins that arise upon HCV-polyprotein processing are indicated.

 The next step in our approach (Figure 1) was the identification of highly conserved epitopes. In order to do so, we first carried out a variability analysis of a multiple sequence alignment (MSA) of HCV-polyproteins using Shannon entropy, *H* (details in Section 2). The MSA included HCV polyproteins from all 6 genotypes; however, genotype 1 was the most represented, with 501 sequences (Table 1). Subsequently, we masked in an HCV-polyprotein reference sequence (AN: AAA45676.1) all variable sites within the MSA with *H* > 0.5. We mapped variability onto this sequence rather than the artificial conserved sequence for two reasons: first, conserved sequences derived upon a MSA tend to be too long and contain stretches of amino acids not present in any real sequence, and secondly, AAA45676.1 has long been used as a reference sequence to design overlapping peptides and check T-cell responses. HCV-polyprotein AAA45676.1 with the variable sites masked is depicted in Figure 2 showing the location of the mature proteins. We used this sequence to discard those epitopes that have any residue, including those flanking the C-terminus, with *H* > 0.5. The residue flanking the C-terminus of the CTL epitope is a determinant for proteasome cleavage, and mutations in that residue can abrogate T-cell recognition [47]. Thus, we also required that residue to be invariant. Only 17 epitopes meet these criteria and are shown in Table 2. That such a few peptides have all their residues with entropy *H* ≤ 0.5 reflects the large genomic plasticity of HCV. 

**Figure 3:**
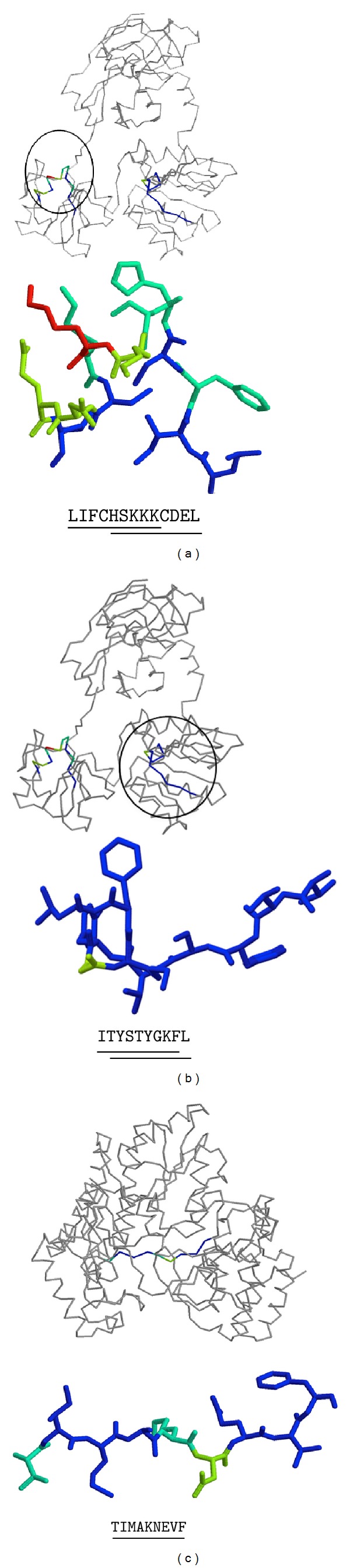
Three-dimensional structure mapping of HCV-specific invariant CTL epitopes. HCV fragments LIFCHSKKKCDEL and ITYSTYGKFL map onto two different regions of the 3D structure of HCV NS3 helicase (PDB: 2F55), (a) and (b), respectively. Each of these two fragments encompasses two overlapping HCV-specific epitopes. The HCV epitope TIMAKNEVF maps onto HCV polymerase (PDB: 2BRK) (c). Residue color used in the illustrations is related to their solvent accessibility and goes from blue for buried residues to red for accessible residues. Overlapping HCV-specific CTL epitopes are shown underlined. Solvent accessibility was calculated and mapped onto the 3D structures as indicated in Section 2. The figures were rendered using Rasmol [48].

**Figure 4:**
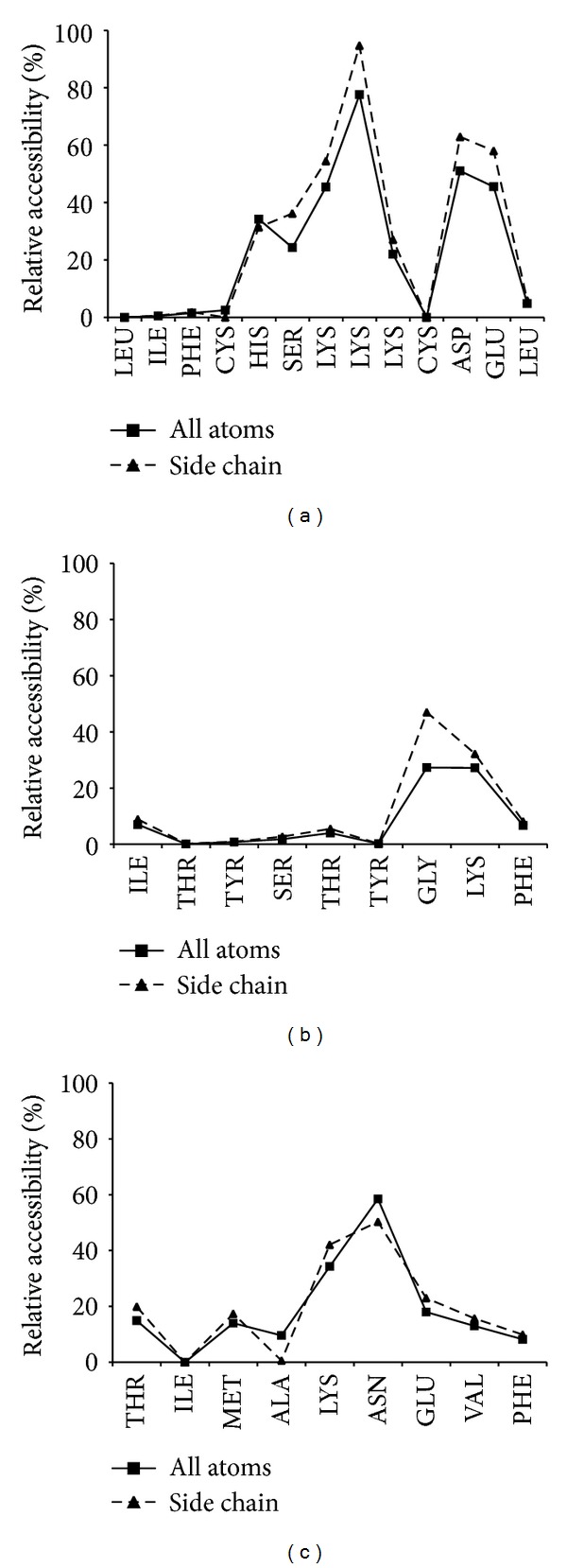
Solvent accessibility of HCV-specific epitopes with counterpart protein 3D structures. The figure depicts a plot of the relative solvent accessibility (%) (*y*-axis) of each of the residues (*x*-axis) in the HCV fragments LIFCHSKKKCDEL (a), ITYSTYGKFL (b) and TIMAKNEVF (c). We plotted both solvent accessibility considering all atoms (squares) and just residue side chains (triangles). Solvent accessibility was calculated from the relevant protein 3D structures using NACCESS [36].

In general, most of the invariant epitopes reside in the CAPSID and CORE proteins of HCV. One reason for this preferred location is that these two proteins seem to be the most conserved. However, regardless of such conservation, we recently showed that proteins located at the N-terminus of polyproteins bear more epitopes than expected for the size because they are preferentially translated [25]. To find structure-function determinants for the conservation of these particular 17 epitopes, we identified three-dimensional structures of HCV proteins bearing the identified epitopes (Table 2) and mapped them onto the 3D structure (Figure 3) (see details in Section 2). We could only map 5 of the 17 conserved epitopes: four of them, LIFCHSKKK, HSKKKCDEL, ITYSTYGKF, and TYSTYGKFL mapped onto two different domains of the NS3 helicase (Figures 3(a) and 3(b)), while one, TIMAKNEVF, mapped onto the RNA polymerase of HCV (Figure 3(c)). Note that the epitopes LIFCHSKKK and HSKKKCDEL overlap and so the epitopes ITYSTYGKF and TYSTYGKFL. Interestingly, all these epitopes bear a large proportion of residues that are hydrophobic and buried in the 3D structure (shown in blue in Figures 3(a), 3(b), and 3(c)). The relative accessibility of each of the epitope residues is shown in Figure 4. For example, the fragment ITYSTYGKFL (it contains two overlapping epitopes) and the epitope TIMAKNEVF only contain residues that are completely buried or semiburied and many are hydrophobic (Figures 3 and 4). These residues form part of the protein hydrophobic core and are key for protein stability. Therefore, the selected epitopes are conserved as they comprise residues involved in protein stability.

Because we aimed to set the basis for a broadly protective epitope-based vaccine against HCV, our next move was to be able to compute the expected population protection coverage (PPC) of any given set of epitopes, defined as the percentage of the population that would be able to elicit a T-cell response against any of them. To this end, we first predicted the binding of each conserved epitope to 55 HLA I molecules for which we have suitable predictors (details in Section 2) thus obtaining predicted epitope HLA I binding profiles. We assumed that any epitope that binds to a given HLA I molecule would be presented and able to elicit a T-cell response. Under this scenario, the HLA I binding profile of a given epitope determines its potential PPC, and we computed it using genetic HLA I frequencies for 5 ethnic groups in the American population [33] (see Section 2 for a detailed explanation). In Table 2, we show the predicted HLA I binding profiles of the 17 conserved HCV-specific epitopes. With this data, we then aimed to identify epitope combinations reaching a 95% PPC in all five ethnic groups being considered, using the method, described in Section 2. This method does not guarantee any multispecificity, as it only seeks for epitope combinations reaching a target PPC. Multispecificity is a common scenario in the course of natural infection as any given individual will elicit T-cell responses against at least two or three immunodominant pathogen-specific epitopes [49]. 

**Figure 5:**
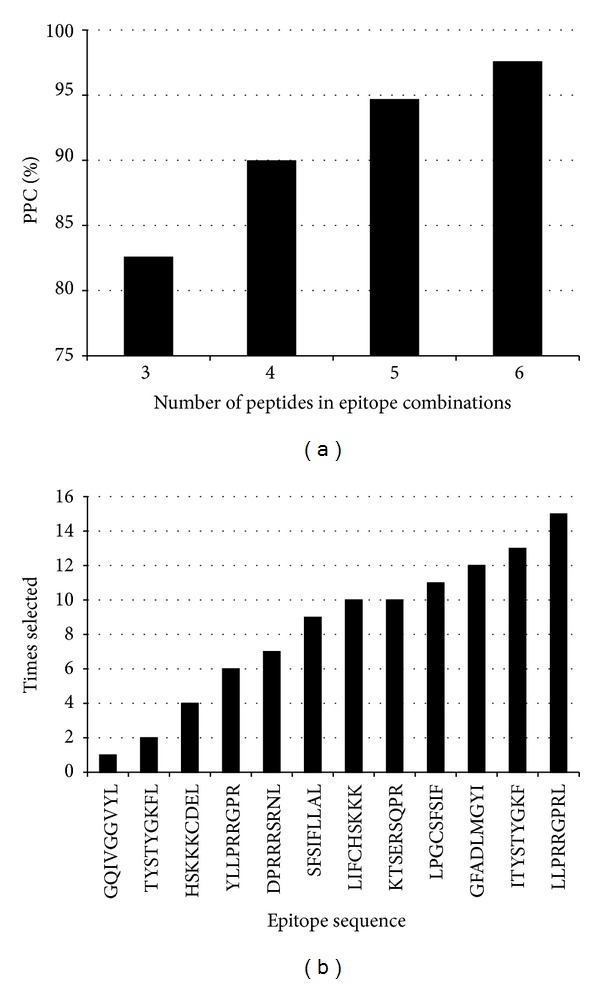
Analysis of HCV-specific CTL epitope set selection. (a) The figure depicts the largest PCC that was reached by HCV-specific CTL epitope combinations containing 3, 4, 5, and 6 epitopes (optimal combinations). As shown, with just 3 epitopes it is possible to reach a PPC ≥ 80% and 6 epitopes were required to reach a PPC ≥ 95%. (b) We found 15 distinct combinations of 6 epitopes each reaching a PPC ≥ 95% within the 17 invariant HCV-specific CTL epitopes. In the figure, we depicted the sequence of peptides (*x*-axis) that were included in the mentioned epitope combinations and the times that were included (*y*-axis).

We found that a PPC > 80% can be reached with just 3 epitopes, LLPRRGPRL, KTSERSQPR/LIFCHSKKK (both epitopes have the same HLA I binding profile), and LPGCSFSIF (Figure 5(a)). This is not surprising as these epitopes seem to target 3 mayor supertypes: A2, A3, and B7 [50–52]. Note, however, that we predicted peptide binding to MHC I molecules beyond predefined HLA I supertypes. To reach a PPC ≥ 95% however, we found that 6 epitopes were required. Moreover, we found that there were 15 different 6-epitope combinations, differing in at least one epitope, providing a PPC ≥ 95%. The largest PPC reached by one of these epitope combinations was of 97% and included the following peptide sequences: LLPRRGPRL, GFADLMGY, KTSERSQPR/LIFCHSKKK, SFSIFLLAL, LPGCSFSIF, and ITYSTYGKF. All 6-epitope combinations reaching PPC ≥ 95% are provided as supplementary data in Additional File S3. All these epitopes combinations differ one from each other in at least one epitope. We also analyzed what epitopes were actually included in the resulting solutions. Of all the epitopes in Table 2, only the first 12 were included in at least one of the epitope combinations reaching PPC ≥ 95%, while the last 5 were never included. The frequency at which the peptides were included in these combinations is shown in Figure 5(b) and varied from 15 times for peptide LLPRRGPRL (it was included in each of the epitope combinations reaching PPC ≥ 95%) to only once for GQIVGGVYL.

Overall, the results shown here indicate that only a handful of conserved HCV-specific epitopes would be needed to elicit CD8 T-cell responses in any individual regardless of their genetic background. We have preliminary data corroborating these predictions. Thus, we have consistently detected responses on T cells from naïve people primed and expanded using dendritic cells loaded with HCV-peptide pools representing single 6-epitope combinations of PPC ≥ 95% (data not shown). However, we only analyzed the responses in a few individuals and more experiments are underway. It is also important to highlight that our results only provide conserved CTL epitope solutions for a potential epitope-based vaccine against HCV to be broadly protective. We know that the eventual development of an epitope-based vaccine against HCV is a complex task that will require optimizing peptide-epitope assembling and vaccine delivery [53]. Moreover, such vaccine will also need to incorporate Th epitopes as well as signals/adjuvants for alerting the innate immune system and initiate an adaptive response. 

**Figure 6:**
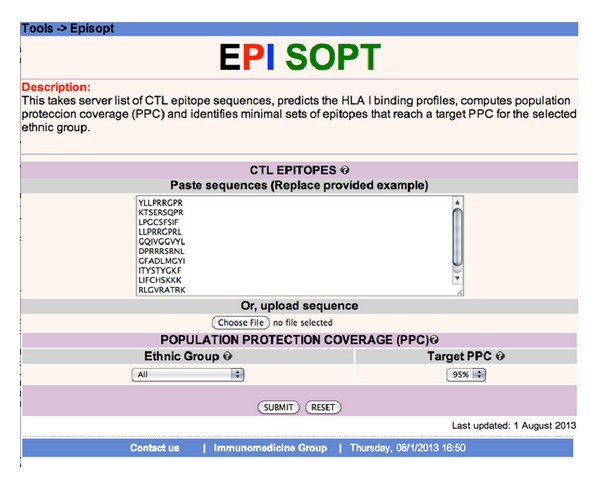
EPISOPT web page. The figure shows the input page of EPISOPT. Currently, users enter a list of CTL epitope sequences and select a target PPC and population group. On submission, EPISOPT will find minimal epitope combinations reaching the desired PPC in the selected population group.

 While in this study we focused on HCV, the same approach could easily be extended to other pathogens; there are thousands of CTL epitopes readily available for many other pathogens. To facilitate that end, we introduced the EPISOPT server (http://imed.med.ucm.es/Tools/episopt.html). The EPISOPT server (Figure 6) implements the steps in our approach consisting of predicting epitope HLA I binding profiles and identifying minimal sets of epitopes reaching a determined PPC. In the future, we will enhance EPISOPT with sequence variability analyses to select invariant epitopes. EPISOPT is somewhat similar to another online tool developed by Bui et al. [54] available at http://tools.immuneepitope.org/tools/population/iedb_input. Both tools implement a similar method to compute PPC. However, unlike EPISOPT, Bui et al. [54] tool requires entering the HLA I binding profiles of epitopes as input—it does not predict them—and it does not find epitope combinations reaching a given PPC. We are hopeful that in time, the results and methods derived from this work will make a significant contribution to the design of epitope-based vaccines.

## Supplementary Material

Additional File S1, HCV-specific T cell epitopes. The epitopes were collected from the EPIMHC [22] and IEDB [23] databases. They all were reported to be targeted in the course of a natural infection by HCV in humans and have nine residues.
Additional File S2, Multiple sequence alignment of HCV-polyproteins. The alignment was built upon 684 entire HCV-polyproteins using MUSCLE [24] with default settings.
Additional File S3, CTL epitopes from HCV reaching PPC ≥ 95%. File shows epitope combinations that were found to reach a PPC ≥ 95% for 5 ethnic groups. Any epitope combination differs from all the others in at least one epitope sequence.

## Figures and Tables

**Table 1:** HCV genotype of polyproteins used in this study.

Genotype	Sequences
1	501
2	50
3	7
4	34
5	2
6	61
Unassigned*	29

*Sequences could not be readily classified onto any of the 6 major genotypes. Genotypes were assigned using MuLDAS (http://www.muldas.org/MuLDAS/).

**Table 2:** Selected conserved HCV-specific T-cell epitopes with predicted HLA I binding profiles.

Epitope	Ct	Region	N	3D	3D-N	PPC	HLA I binding profile
KTSERSQPR	G	CAPSID	71	NA	—	32.2%	A0301 A1101 A3101 A3301 A6801
LIFCHSKKK	C	HELICc	1391	2F55∣A	365–373	32.2%	A0301 A1101 A3101 A3301 A6801
LLPRRGPRL	G	CAPSID	36	1XCQ∣P	—	28.2%	A0201 A0203 A0205 A0206 C0102
LPGCSFSIF	L	CORE	169	NA	—	27.8%	B0702 B1502 B1508 B3501 B5301 B5401
DPRRRSRNL	G	CAPSID/CORE	101	NA	—	26.9%	B0702 B0801 B5101 B5102 B5103 B5301 B5401
GFADLMGYI	P	CORE	129	NA	—	6.3%	A0202 A0203 A0205 A6802 B3801
NLPGCSFSI	F	CORE	168	NA	—	5.9%	A0205 C0102
SFSIFLLAL	L	CORE	173	NA	—	5.5%	A2402
TYSTYGKFL	A	DEXDC	1292	2F55∣A	266–274	5.5%	A2402
ITYSTYGKF	L	DEXDC	1291	2F55∣A	265–273	2.7%	B1516 B1517 B5702 B5801
HSKKKCDEL	A	HELICc	1395	2F55∣A	369–377	2.5%	B0801
YLLPRRGPR	L	CAPSID	35	1XCQ∣P	—	1.4%	A3301 A6601 A6801
GQIVGGVYL	L	CAPSID	28	1XCQ∣P	—	0.4%	A0206 A0214 B1510 B4002
TIMAKNEVF	C	RNADP	2587	2BRK∣A	137–145	0	B1513 B5702
GPRLGVRAT	R	CAPSID	61	NA	—	0	
RLGVRATRK	T	CAPSID	63	NA	—	0	
STGLIHLHQ	N	NS1	686	NA	—	0	

Epitope: amino acid sequence of the epitope; Ct: residue that flanks the C-terminal end of the epitope; Region: protein/domain in HCV polyprotein bearing the epitope; N: position in HCV polyprotein of the epitope N-terminal residue; 3D: PDB code with 3D structure of protein encompassing the epitope; 3D-N: location of the epitope in the 3D structure; PPC: population protection coverage; HLA I binding profile: HLA I molecules predicted to present the epitope. All epitope residues and c-terminal flanking residues have *H* ≤ 0.5. PPC values equal to the lower value computed with the gene frequencies corresponding to 5 ethnic populations [33]. PDB 1XCQ∣P corresponds to a peptide from HCV capsid bound to an antibody Fab; hence we do not show the location of the epitope in the 3D structure. NA: PDB not available.
